# Metabolic associated fatty liver disease and sarcopenia additively increase mortality: a real-world study

**DOI:** 10.1038/s41387-023-00250-6

**Published:** 2023-11-15

**Authors:** Qianwen Zhao, Yifan Yin, Yunlei Deng

**Affiliations:** 1 Department of Gastroenterology & Hepatology, West China Hospital, Sichuan University, Chengdu, Sichuan 610041 China; 2 Sichuan University-University of Oxford Huaxi Joint Centre for Gastrointestinal Cancer, Frontiers Science Center for Disease-Related Molecular Network, West China Hospital, Sichuan University, Chengdu, Sichuan 610200 China; 3 Department of Nephrology, The Third People’s Hospital of Chengdu, Affiliated Hospital of Southwest Jiaotong University, 82#Qinglong Street, Qingyang District, Chengdu, 610031 China

**Keywords:** Metabolic syndrome, Nutrition, Risk factors

## Abstract

**Background and aims:**

Sarcopenia is associated with worse prognosis for non-alcoholic fatty liver disease (NAFLD). However, disease progression in the MAFLD-related sarcopenia is largely unknown. We aimed to clarify the relationship between MAFLD and/or sarcopenia with mortality and liver fibrosis in the real world.

**Methods:**

A total of 13,692 individuals were selected from the third National Health and Nutrition Examination Surveys and linked mortality until December 2019. MAFLD is diagnosed based on a radiologically diagnosed hepatic steatosis and the presence of any one of the following three conditions: overweight/obesity, diabetes mellitus (DM), or metabolic dysregulation. Sarcopenia is defined by weight-adjusted skeletal muscle mass.

**Results:**

The mean age was 43.7 ± 15.97 years, and 47.3% of the individuals were male. MAFLD was diagnosed in 4207/13,692 (30.73%) participants, and the proportion of sarcopenic was 19.42% amongst subjects with MAFLD. The mean follow-up duration was of 23.7 ± 7.62 years. MAFLD (aHR 1.152, 95% CI 1.070–1.241) and sarcopenia (aHR 1.123, 95% CI 1.042–1.210) were related to increased all-cause mortality in MAFLD after adjustment for age, sex, race, marital status, education, and smoking. Stratified analysis revealed that MAFLD and sarcopenia additively increased the risk of mortality (aHR 1.247, 95% CI 1.132–1.373) and liver fibrosis (aOR 2.296, 95% CI 1.718–3.069 assessed by NFS score >0.676; aOR 2.218, 95% CI 1.788–2.752 assessed by FIB-4 score >1.3) in fully adjusted models (*P* < 0.001 for all).

**Conclusion:**

Sarcopenia in individuals with MAFLD portends increased mortality and significant liver fibrosis. Novel therapeutic strategies targeting at increasing skeletal muscle mass should be explored for patients with MAFLD.

## Introduction

Metabolic dysfunction-associated fatty liver disease (MAFLD) is a new nomenclature proposed by an international consensus in 2020 that highlights the metabolism dysregulation that accompany fatty liver [1]. Unlike non-alcoholic fatty liver disease (NAFLD), exclusion of secondary causes is not required for the diagnosis of MAFLD. Compared with NAFLD, MAFLD is related to greater risk of advanced fibrosis, all-cause mortality, and cardiovascular disease-related mortality [2, 3]. Sarcopenia is a geriatric disease characterized by a gradual loss of skeletal muscle mass and muscle function [4]. With the growing prevalence globally, sarcopenia and low muscle mass are reported to significantly increase mortality in both the elderly population and young adults [5–7]. Therefore, the presence of sarcopenia should be considered in patients with MAFLD since they are with high possibility for disease deterioration. As reported previously, sarcopenia was associated with a higher risk of mortality in population with NAFLD [8]. Another Korean population-based study found that subjects with MAFLD and sarcopenia had higher risks of liver fibrosis and cardiovascular risk [9]. As sarcopenia contributes to higher risk of mortality, coexisting MAFLD and sarcopenia could be associated with a higher mortality and fibrosis risk. However, there is still limited evidence to correlate MAFLD-associated sarcopenia with all-cause and cause-specific mortality.

Herein, we used a population-based data from the National Health and Nutrition Examination Surveys (NHANES) and the National Center for Health Statistics (NCHS) to investigate the independent relationship between the presence of MAFLD and/or sarcopenia with all-cause and cause-specific mortality in the general population. We also assessed whether the stratification of individuals with MAFLD using sarcopenia status could identify subgroups with different outcomes of liver fibrosis.

## Methods

### Patient inclusion

This study was performed within the third National Health and Nutrition Examination Surveys 1988–1994 (NHANES III), a periodic survey conducted by the National Center for Health Statistics of the Centers for Disease Control and Prevention of the United States. This national dataset was designed to study participants’ health and nutritional status in the United States. The NHANES collected detailed information on health and nutrition by interview, physical examination, and a battery of clinical measurements and tests from all members. The National Center for Health Statistics Research Ethics Review Board approved the NHANES protocol and informed consent was obtained from all subjects. The detailed dataset and further information are available at https://www.cdc.gov/nchs/nhanes/about_nhanes.htm. Individuals who were part of NHANES III (1988–1994) with available data on MAFLD and sarcopenia were eligible for inclusion. The exclusion criteria were age <20 years, missing data on BIA, liver function, and liver ultrasound.

### Diagnosis of MAFLD and definition of advanced fibrosis

The diagnosis of MAFLD is based on ultrasound defined hepatosteatosis and the presence of any one of the following three conditions, namely overweight/obesity, diabetes mellitus (DM), and metabolic dysregulation [1]. Metabolic dysregulation was defined as the presence of no less than two the following conditions: (a) Waist circumference ≥102 cm in men and 88 cm in women. (b) Arterial blood pressure ≥130/85 mmHg or taking with anti-hypertension drugs. (c) Serum triglycides ≥1.70 mmol/L or specific drug treatment. (d) Serum high-density lipoprotein cholesterol <1.0 mmol/L for males and <1.3 mmol/L for females. (e) Prediabetes (i.e., fasting glucose levels 5.6 to 6.9 mmol/L, or 2-h postload glucose levels 7.8–11.0 mmol/L or glycated hemoglobin 5.7% to 6.4%). (f) Homeostasis model assessment-insulin resistance (HOMA-IR) score ≥2.5. (g) C-reactive protein (CRP) level >2 mg/L. The hepatic fibrosis was assessed by fibrosis-4 index (FIB-4) or NAFLD fibrosis score (NFS). The equation of FIB-4 score: FIB-4 = (age [years] × AST [U/L])/(platelet [10^9^/L] × $\surd ({\rm{ALT}}[{\rm{U}}/{\rm{L}}])$ [10]. The equation of NFS score: The NFS = −1.675 + 0.037 × age (years) + 0.094 × BMI (kg/m^2^) + 1.13 × impaired fasting glucose/diabetes (yes = 1, no = 0) + 0.99×AST/ALT − 0.013 × platelet (×10^9^/L) − 0.66 × albumin (g/dl) [11]. According to the FIB-4 and NFS scores, participants were categorized into low fibrosis, intermediate fibrosis, and significant fibrosis groups, naming FIB-4 < 1.30/NFS < −1.455, FIB-4 1.30 to 2.67/NFS −1.455 to 0.676, FIB-4 > 2.67/NFS > 0.676), respectively.

### Definition of sarcopenia

Sarcopenia is defined by weight-adjusted skeletal muscle mass, which could be evaluated by bioelectrical impedance analysis (BIA), height, sex, etc. Skeletal muscle mass (kg) = ([height^2^/BIA-resistance × 0.401] + [sex × 3.825] + [age × –0.071]) + 5.102 (height [centimeters]; BIA resistance [ohms]; sex [female = 0 and male = 1]; and age [years]) [12]. The Skeletal muscle index (SMI) was calculated by dividing skeletal muscle mass (kg) by body mass (kg) and multiplying it by 100%. Sarcopenia was defined as SMI over one standard deviation below the sex specific, young adult (age 20–39) means: 37.0% in men and 28.0% in women [13].

### Follow-up and mortality data

Information regarding vital status was obtained from the National Death Index and provided by the National Center for Health Statistics (NHCS) which contained complete data until December 2019.

### Demographic variables

From the dataset, we obtained the following demographic variables: age, sex, race, education, income, marital status, smoking history, current smoking status, body mass index (BMI), insulin resistance, history of hypertension, CVD, and DM. Overweight or obesity is defined as BMI ≥ 25 kg/m^2^. CKD is defined as either an estimated glomerular filtration rate (eGFR) of ≤60 mL/min/1.73 m^2^ or the presence of albuminuria. The CKD-EPI equation calculator was used to calculate estimating glomerular filtration rate (eGFR), eGFR = 141 × min(Scr/κ, 1)α × max(Scr/κ, 1) − 1.209 × 0.993Age × 1.018 [if female] × 1.159 [if African American] (serum creatinine = mg/dL, κ = 0.7 (females) or 0.9 (males), *α* = −0.329 (females) or −0.411 (males), min = indicates the minimum of SCr/κ or 1, max = indicates the maximum of SCr/κ or 1, age = years) [14]. The definition of hypertension is systolic blood pressure (SBP) ≥ 140 mmHg and/or diastolic blood pressure (DBP) ≥ 90 mmHg or taking antihypertensive medications. Diabetes is defined as a physician diagnosis of diabetes or the use of antidiabetic medications.

### Laboratory parameters

Laboratory measurements included aspartate aminotransferase (AST), alanine transaminase (ALT), glycated hemoglobin (HbA1c), total cholesterol (TC), high-density lipoprotein cholesterol (HDL-C), and blood creatinine. All biochemical assessments were performed by standard laboratory methods.

### Statistical analysis

Continuous variables are described as the mean ± standard deviation. Categorical variables are described as numbers (percentages). The Kruskal-Wallis test, chi-square test, Fisher’s exact test, Cox regression, and logistic regression analysis were used to assess significant differences and risk factors with SPSS (IBM, version 26.0). Microsoft Excel (version 16.48), and Microsoft PowerPoint (version 16.48) were used to collect data and generate figures. *P* < 0.05 was considered statistically significant.

## Results

### Baseline characteristics

29,314 participants from the NHANES III cohort (1988–1994) with available ultrasonography and laboratory results were included in the study. Of them, 12,284 were excluded for age <20, 1192 for missing data on AST or ALT, 1982 for lack of liver ultrasound data, and 164 for missing data on BIA, leaving 13,692 participants for analysis. The mean age of the population was 43.7 ± 15.97 years, 6473/13,692 (47.3%) were male and the mean BMI was 27.3 ± 5.90 kg/m^2^. MAFLD was diagnosed in 4207/13,692 (30.73%) participants and sarcopenia in 5804/13,692 (42.39%) among the overall population, resulting in the following distribution of reciprocally independent groups: MAFLD [-]/sarcopenia [-] (46.30%), MAFLD [+]/sarcopenia [-] (11.31%), MAFLD [-]/sarcopenia [+] (22.97%) and MAFLD [+]/sarcopenia [+] (19.42%). Detailed baseline information of these groups are shown in Table 1.

**Table 1:** Participants’ characteristics stratified for sarcopenia and MAFLD status.

	MAFLD-/SARCOPENIA- *N* = 6340	MAFLD + /SARCOPENIA- *N* = 1548	MAFLD-/SARCOPENIA+ *N* = 3145	MAFLD + /SARCOPENIA+ *N* = 2659
Age (years)	38.35 ± 14.53	43.69 ± 14.60	48.23 ± 16.44	51.14 ± 14.80
Male	3302 (52.1%)	954 (61.6%)	1063 (33.8)	1154 (43.4)
Race				
Non-hispanic black	2476 (39.1%)	465 (30.0%)	1095 (34.8%)	958 (36.0%)
Non-hispanic white	1838 (29.0%)	377 (24.4%)	1166 (37.1%)	651 (24.5%)
Mexican-American	1716 (27.1%)	619 (40.0)	791 (25.2%)	965 (36.3)
Other	310 (4.9%)	87 (5.6%)	93 (3.0%)	85 (3.2%)
Education				
≤ High school degree	4155 (65.5%)	1169 (75.5%)	2272 (72.2%)	2076 (78.1%)
> High school degree	2185 (34.5%)	379 (24.5%)	873 (27.8%)	583 (21.9%)
Poverty income ratio				
<1	1802 (28.4%)	532 (34.4%)	968 (30.8%)	855 (32.2%)
1≤ and <5	3925 (61.9%)	883 (57.0%)	1893 (60.2%)	1616 (60.8%)
≥5	613 (9.7%)	133 (8.6%)	284 (9.0%)	188 (7.1%)
Current smoking	2124 (33.5%)	435 (28.1%)	813 (25.9%)	541 (20.3%)
Smoking history	3273 (51.6%)	847 (54.7%)	1584 (50.4%)	1367 (51.4%)
Diabetes	196 (3.1%)	193 (12.5%)	221 (7.0%)	413 (15.5%)
Hypertension	917 (14.5%)	406 (26.2%)	991 (31.5%)	1121 (42.2%)
CVD history	209 (3.3%)	82 (5.3%)	221 (7.0%)	290 (10.9%)
Heart Attack	65 (1.0%)	23 (1.5%)	66 (2.1%)	87 (3.3%)
Congestive Heart Diseases	119 (1.9%)	51 (3.3%)	121 (3.8%)	165 (6.2%)
Chronic kidney Disease	89 (1.4%)	36 (2.3%)	111 (3.5%)	140 (5.3%)
Body mass index (kg/m^2^)	23.93 ± 3.49	27.07 ± 3.65	29.25 ± 5.71	33.00 ± 6.31
Skeletal muscle mass (kg)	26.07 ± 6.61	28.416 ± 6.95	19.31 ± 9.77	23.24 ± 9.77
Skeletal muscle index (%)	38.3 ± 6.38	37.09 ± 5.68	23.44 ± 10.15	25.44 ± 9.10
AST (U/L)	21.76 ± 15.90	26.06 ± 20.43	20.54 ± 11.67	25.11 ± 18.91
ALT (U/L)	16.82 ± 15.69	25.10 ± 23.41	15.75 ± 11.55	23.62 ± 22.06
HDL-C (mmol/L)	1.39 ± 0.40	1.19 ± 0.38	1.35 ± 0.41	1.18 ± 0.34
Triglycerides (mmol/L)	1.26 ± 0.88	2.25 ± 2.05	1.59 ± 1.12	2.17 ± 1.60
HbA1c (%)	5.26 ± 0.77	5.74 ± 1.41	5.56 ± 1.07	6.02 ± 1.50
Creatinine (μmol/L)	94.34 ± 30.08	96.26 ± 22.87	93.37 ± 31.87	94.50 ± 33.78
eGFR (mL/min/1.73m^2^)	83.73 ± 17.06	80.58 ± 17.97	77.62 ± 20.22	75.21 ± 18.38
HOMA-IR	2.30 ± 6.52	4.66 ± 8.94	3.50 ± 10.16	6.24 ± 10.98

Categorical values are shown as *n* (%). Continuous variables are shown as mean ± standard deviation.

*ALT* alanine aminotransferase, *AST* aspartate aminotransferase, *CVD* cardiovascular disease, *eGFR* estimated glomerular filtration rate, *HbA1c* glycated hemoglobin, *HDL-C* high-density lipoprotein cholesterol, *HOMA-IR* homeostatic model assessment-insulin resistance, *NAFLD* non-alcoholic fatty liver disease.

### Mortality according to MAFLD and sarcopenic status

During the follow-up of 23.7 ± 7.62 years, there were 4883 deaths in total. Among them, there were 1328 cardiovascular-related mortality, 1178 cancer-related death and 209 diabetes-related mortality (*n* = 209). MAFLD (aHR 1.152, 95% CI 1.070–1.241) and sarcopenia (aHR 1.123, 95% CI 1.042–1.210) were independently and simultaneously associated with increased all-cause mortality in the fully adjusted models (Table 2). Furthermore, to clarify whether the existence of MAFLD or sarcopenia increased the hazard for mortality, we categorized the cohort into four groups: (MAFLD [-]/sarcopenia [-], MAFLD [+]/sarcopenia [-], MAFLD [-]/sarcopenia [+] and MAFLD [+]/sarcopenia [+]). Compared with patients without MAFLD and sarcopenia, patients with MAFLD and sarcopenia were with higher risk of all-cause mortality (aHR 1.247, 95% CI 1.132–1.373) in the fully adjusted models. However, in the MAFLD [+]/sarcopenia [-] and MAFLD [-]/sarcopenia [+] groups, we could not demonstrate an increased risk for all-cause mortality compared with the MAFLD [-]/sarcopenia [-] group (aHR 1.076, 95% CI 0.952–1.217 and aHR 1.055, 95% CI 0.959–1.161, respectively) (Table 3).

**Table 2:** All-cause mortality risk for the presence of MAFLD and sarcopenia.

	HR	95% CI	*P*
Model 1			
MAFLD	1.115	1.053–1.182	0.029
SARCOPENIA	1.095	1.032–1.163	0.030
Model 2			
MAFLD	1.152	1.070–1.241	0.000
SARCOPENIA	1.123	1.042–1.210	0.002

Results were obtained with Cox proportional hazards and given as HR with 95% CI for all-cause mortality as outcome. Results were adjusted for age and sex (model 1) and in addition for race, marital status, education, and smoking (model 2).

*CI* confidence interval, *HR* hazard ratio, *MAFLD* metabolic dysfunction associated fatty liver disease.

**Table 3:** All-cause mortality risk for the four mutually exclusive groups based on MAFLD and sarcopenia.

	HR	95% CI	*P*
Model 1			
MAFLD-/SARCOPENIA-	**Reference**		
MAFLD + /SARCOPENIA-	1.033	0.936–1.141	0.517
MAFLD-/SARCOPENIA+	1.045	0.968–1.128	0.262
MAFLD + /SARCOPENIA+	1.170	1.085–1.262	0.000
Model 2			
MAFLD-/SARCOPENIA-	**Reference**		
MAFLD + /SARCOPENIA-	1.076	0.952–1.217	0.238
MAFLD-/SARCOPENIA+	1.055	0.959–1.161	0.271
MAFLD + /SARCOPENIA+	1.247	1.132–1.373	0.000

Results were obtained with Cox proportional hazards and given as HR with 95% CI for all-cause mortality as outcome. Results were adjusted for age and sex (model 1) and in addition for race, marital status, education, and smoking (model 2).

*CI* confidence interval, *HR* hazard ratio, *MAFLD* metabolic dysfunction associated fatty liver disease.

Concerning cause-specific mortality, cardiovascular disease-related mortality was comparable for the MAFLD or sarcopenia group independently (Table 4). However, in multivariable analysis, the MAFLD [+]/sarcopenia [+] group was with a 22.9% increase in cardiovascular disease-related mortality risk compared with the MAFLD [-]/sarcopenia [-] group (aHR: 1.229 95% CI: 1.019–1.482) (Table 5).

**Table 4:** CVD-related mortality risk for the presence of MAFLD and sarcopenia.

	HR	95% CI	*P*
Model 1			
MAFLD	1.101	0.986–1.229	0.087
SARCOPENIA	1.052	0.938–1.179	0.388
Model 2			
MAFLD	1.105	0.959–1.273	0.166
SARCOPENIA	1.137	0.985–1.311	0.079

Results were obtained with Cox proportional hazards and given as HR with 95% CI for all-cause mortality as outcome. Results were adjusted for age and sex (model 1) and in addition for race, marital status, education, and smoking (model 2).

*CI* confidence interval, *HR* hazard ratio, *MAFLD* metabolic dysfunction associated fatty liver disease.

**Table 5:** CVD-related mortality risk for the four mutually exclusive groups based on MAFLD and sarcopenia.

	HR	95% CI	*P*
Model 1			
MAFLD-/SARCOPENIA-	**Reference**		
MAFLD + /SARCOPENIA-	1.122	0.931–1.353	0.225
MAFLD-/SARCOPENIA+	1.033	0.892–1.196	0.665
MAFLD + /SARCOPENIA+	1.117	0.965–1.292	0.138
Model 2			
MAFLD-/SARCOPENIA-	**Reference**		
MAFLD + /SARCOPENIA-	1.141	0.904–1.441	0.267
MAFLD-/SARCOPENIA+	1.350	0.945–1.363	0.175
MAFLD + /SARCOPENIA+	1.229	1.019–1.482	0.031

Results were obtained with Cox proportional hazards and given as HR with 95% CI for all-cause mortality as outcome. Results were adjusted for age and sex (model 1) and in addition for race, marital status, education, and smoking (model 2).

*CI* confidence interval, *HR* hazard ratio, *MAFLD* metabolic dysfunction associated fatty liver disease.

In addition, regarding diabetes-related mortality, individuals with MAFLD had a 158.9% higher diabetes-related mortality than those without MAFLD in the age and gender adjusted model (HR: 2.589, 95% CI: 1.960–3.421). In the fully adjusted model, MAFLD was significantly associated with an increased risk for diabetes-related mortality (aHR: 2.532, 95% CI: 1.759–3.645). Individuals with sarcopenia had increased risk of diabetes-related mortality (aHR: 1.657; 95% CI: 1.226–2.239) in the age and sex adjusted model. However, sarcopenia failed to maintain this association in the fully adjusted model (aHR: 1.444, 95% CI: 0.988–2.112) (Table 6). Furthermore, compared with individuals without MAFLD and sarcopenia, individuals with MAFLD were significantly associated with a higher risk of diabetes-related mortality both with and without sarcopenia in the fully adjusted model. However, for the MAFLD [+]/sarcopenia [+] group, this association was failed to be maintained in the fully adjusted model (Table 7). In addtion, as shown in Tables S1–S2, individuals with MAFLD or sarcopenia, or in any mutually exclusive group were not significantly related to a higher risk of cancer-related mortality.

**Table 6:** Diabetes-related mortality risk for the presence of MAFLD and sarcopenia.

	HR	95% CI	*P*
Model 1			
MAFLD	2.589	1.960–3.421	0.000
SARCOPENIA	1.657	1.226–2.239	0.001
Model 2			
MAFLD	2.532	1.759–3.645	0.000
SARCOPENIA	1.444	0.988–2.112	0.058

Results were obtained with Cox proportional hazards and given as HR with 95% CI for all-cause mortality as outcome. Results were adjusted for age and sex (model 1) and in addition for race, marital status, education, and smoking (model 2).

*CI* confidence interval, *HR* hazard ratio, *MAFLD* metabolic dysfunction associated fatty liver disease.

**Table 7:** Diabetes-related mortality risk for the four mutually exclusive groups based on MAFLD and sarcopenia.

	HR	95% CI	*P*
Model 1			
MAFLD-/SARCOPENIA-	**Reference**		
MAFLD + /SARCOPENIA-	2.241	1.395–3.601	0.001
MAFLD-/SARCOPENIA+	1.166	0.742–1.834	0.506
MAFLD + /SARCOPENIA+	3.245	2.210–4.766	0.000
Model 2			
MAFLD-/SARCOPENIA-	**Reference**		
MAFLD + /SARCOPENIA-	2.905	1.633–5.169	0.000
MAFLD-/SARCOPENIA+	1.228	0.580–1.834	0.479
MAFLD + /SARCOPENIA+	3.023	1.823–5.012	0.000

Results were obtained with Cox proportional hazards and given as HR with 95% CI for all-cause mortality as outcome. Results were adjusted for age and sex (model 1) and in addition for race, marital status, education, and smoking (model 2).

*CI* confidence interval, *HR* hazard ratio, *MAFLD* metabolic dysfunction associated fatty liver disease.

### Significant liver fibrosis risk based on MAFLD and sarcopenic status

**Fig. 1:**
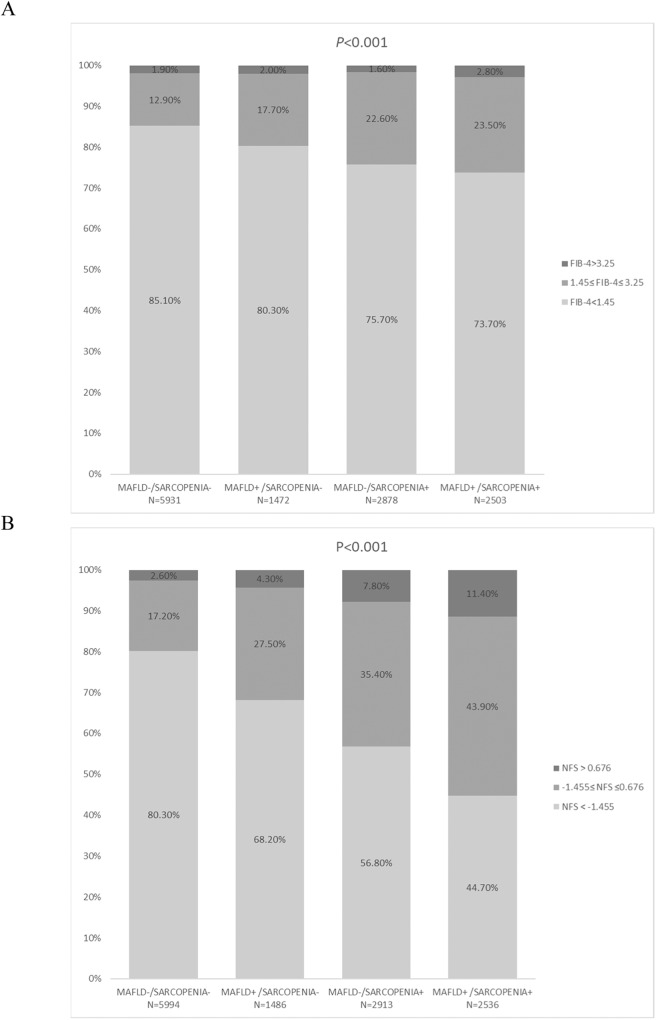
Distribution of fibrosis by FIB-4/NFS categories by MAFLD and sarcopenia categories. When **A** fibrosis-4 index (FIB-4) and **B** non-alcoholic fatty liver disease fibrosis score (NFS) were stratified by risk degree, the degree of fibrotic burden significantly increased from population without MAFLD and sarcopenia, to MAFLD population without sarcopenia and sarcopenic population.

Subjects in the MAFLD [+]/sarcopenia [+] group had the highest rate of advanced fibrosis (2.8% by FIB-4 and 11.4% by NFS). Although MAFLD [-]/sarcopenia [+] has a slightly lower rate of advanced fibrosis evaluated by FIB-4, the overall median and advanced fibrosis in MAFLD [-]/sarcopenia [+] is significantly higher than that of MAFLD [+]/sarcopenia [-]. In the plateau of NFS evaluated liver fibrosis, followed by the MAFLD [-]/sarcopenia [+] group (1.6% by FIB-4 and 7.8% by NFS) and the MAFLD [+]/sarcopenia [-] group (2.0% by FIB-4 and 4.3% by NFS), and the MAFLD [-]/sarcopenia [-] group had the lowest rate of advanced liver fibrosis (1.9% by FIB-4 and 2.6% by NFS) (*P* < 0.001) (Fig. 1).

As there were limited number of subjects with FIB-4 > 2.67 in the dataset, we used FIB-4 > 1.3 and NFS > 0.676 to define significant liver fibrosis [9, 15]. After adjustments for possible confounders (age, sex, race, marital status, education, and current smoking status), subjects with sarcopenia showed significantly higher aORs for significant liver fibrosis, by both FIB-4 and NFS compared with subjects without sarcopenia (all *P* < 0.001) (Tables S3–S4). When compared with the MAFLD [-]/sarcopenia [-] group, the MAFLD [-]/sarcopenia [+] group (aOR = 1.600 by FIB-4; aOR = 1.566 by NFS) and MAFLD [+]/sarcopenia [+] group (aOR = 1.647 by FIB-4; aOR = 2.296 by NFS) were both with higer risk of significant liver fibrosis (all *P* < 0.001). Furthermore, for the MAFLD [+]/sarcopenia [-] group, subjects failed to show a significantly increased risk for fibrosis by both FIB-4 and NFS after adequate adjustment (Tables S5–S6).

## Discussion

In this longitudinal, population-based cohort study, our results supported that the simultaneous existence of MAFLD and sarcopenia was associated with an increased risk of mortality and significant fibrosis. When compared with subjects without MAFLD or sarcopenia, subjects with sarcopenia and MAFLD had a 1.247-fold increased risk of all-cause mortality, whereas non-sarcopenic subjects with MAFLD and sarcopenic subjects without MAFLD did not show an increased risk for mortality. Concerning cause-specific mortality, patients with MAFLD coexisting sarcopenia had an increased risk of CVD- and diabetes-related mortality. In addition, when compared with the MAFLD [-]/sarcopenia [-] group, subjects in MAFLD [-]/sarcopenia [+] group had a 1.65-fold increased risk for significant fibrosis and subjects in MAFLD [+]/sarcopenia [+] group had a 2.218-fold increased risk, whereas subjects in MAFLD [+]/sarcopenia [-] group did not have advanced fibrosis risk. Taken together we have shown convincing evidence supporting the clinical relevance of MAFLD and sarcopenia with poor outcomes.

Up to now, this is the first study which include long-term mortality data stratified by sarcopenia status in population with MAFLD. Different from NAFLD which is controversial about whether it is associated with an increased mortality based on recent studies [3, 8], a few studies have reported that MAFLD was associated with increased all-cause mortality [3, 16, 17]. Recently, Sun et al. and Golabi et al. reported that sarcopenia independently increased overall mortality and cardiac mortality in patient with NAFLD [18, 19]. Liu et al. also found that predicted fat mass and lean mass were independent predictors for overall and cause-specific mortality [20]. These studies indicate that both sarcopenia and MAFLD per se are related to increased risk of mortality, which could explain our findings that sarcopenia in the setting of MAFLD is associated with mortality and sarcopenia should be evaluated for risk stratification of mortality in subjects with MAFLD.

In the past few years, the possible pathogenetic mechanisms linking sarcopenia with fatty liver disease have been widely studied, including insulin resistance, systemic inflammation, insulin resistance, physical inactivity, hormone dysregulation and vitamin D deficiency [21]. The positive association of sarcopenia with prognosis of NAFLD has been supported by several studies. Moon et al. reported that concurrent NAFLD and sarcopenia conferred a 2-fold higher risk of mortality [22]. A study by Kim et al. also concluded that sarcopenia was associated with a higher risk for all-cause, cancer- and diabetes-related mortality in individuals with NAFLD [8]. Currently, there are still limited data about the long-term prognosis of subjects with simultaneous MAFLD and sarcopenia. A recent Korean cross-sectional study by Chun et al. demonstrated that sarcopenic subjects with MAFLD had higher risks of significant liver fibrosis and arteriosclerotic cardiovascular disease (ASCVD) score [9]. Consistent with their conclusion, we also demonstrated that the MAFLD[+]/sarcopenia[+] group had a higher risk of advanced fibrosis by NFS score compared with other groups. However, Chun et al. did not include long-term outcome data such as mortality. The current study compared the all-cause and cause-specific mortality stratified by sarcopenia status in the population with MAFLD, which made it more robust to note the predictive value of concurrent MAFLD and sarcopenia for prognosis. Furthermore, we showed that the risk of CVD- and diabetes-related mortality for participants with MAFLD and coexisting sarcopenia increased significantly. These results suggest that the complication of sarcopenia was associated with a higher mortality risk of MAFLD.

An advantage of our study is that this is the first cohort study to demonstrate long-term mortality for population with MAFLD stratified by sarcopenia status in a representative population-based database with a substantial follow-up period (mean follow-up 23.7 years), which strengthens the results. However, there are some limitations of our study. First of all, we did not include liver-related mortality which is not available in the current form of the NHANES III dataset. Secondly, we only used weight-adjusted skeletal muscle mass (SM/Wt) to define sarcopenia, however, controversy remains regarding whether height- or weight-adjusted skeletal muscle mass (SM/Ht^2^ or SM/Wt) has a better predictive ability for the diagnosis of sarcopenia [23]. Thirdly, liver steatosis in this study was diagnosed by liver ultrasound instead of biopsy, which used to be the gold-standard but not recommended for the diagnosis of fatty liver disease currently. Besides, a limitation of this study is that the NHANES III dataset lacked ethnic-specific criteria for Asian subgroups. Due to this constraint, the criteria for overweight/obesity and prolonged waist circumference developed for Caucasians were applied to all participants, including Asians. This may have introduced bias, as appropriate BMI and waist circumference cutoffs likely vary between different Asian ethnicities based on prior evidence [1]. Lastly, although we adjusted for multiple potentially confounding factors, there may have been other residual effects from other unadjusted factors of the correlation between sarcopenia, MAFLD, and mortality.

In conclusion, based on our results that sarcopenia is related to higher risk of all-cause, CVD-, and diabetes-related mortality in participants with MALFD independent of other confounding risk factors, sarcopenia could additively increase mortality in the setting of MAFLD. Given the importance of skeletal muscle mass in the population with MAFLD, lifestyle interventions to increase skeletal muscle proportion should be emphasized for patients with MAFLD and sarcopenia.

### Supplementary information

Supplementary tables

## Data Availability

The datasets generated during and/or analyzed during the current study are available from the corresponding author on reasonable request.
