# Supplementary material for: Metabolic associated fatty liver disease and sarcopenia additively increase mortality: a real-world study
Source: Nutr Diabetes. 2023 Nov 15;13:21. doi: 10.1038/s41387-023-00250-6 (PMC10651884; doi:10.1038/s41387-023-00250-6)
Supplement: Supplementary file 1 — Supplementary tables [file 41387_2023_250_MOESM1_ESM.docx]

**Supplementary Table legends**

Table S1 Cancer-related Mortality risk for the presence of MAFLD and sarcopenia

Table S2 Cancer-related Mortality risk for the four mutually exclusive groups based on MAFLD and sarcopenia

Table S3 Fibrosis risk by FIB-4 for the presence of sarcopenia

Table S4 Fibrosis risk by NFS for the presence of sarcopenia

Table S5 Fibrosis risk by FIB-4 for the four mutually exclusive groups based on MAFLD and sarcopenia

Table S6 Fibrosis risk by NFS for the four mutually exclusive groups based on MAFLD and sarcopenia

Table S1：Cancer-related Mortality risk for the presence of MAFLD and sarcopenia

|  | HR | 95% CI | *P* |
| --- | --- | --- | --- |
| **Model 1** |  | | |
| MAFLD | 0.968 | 0.859-1.091 | 0.596 |
| SARCOPENIA | 0.957 | 0.848-1.080 | 0.479 |
| **Model 2** |  | | |
| MAFLD | 1.001 | 0.864-1.160 | 0.989 |
| SARCOPENIA | 0.978 | 0.846-1.130 | 0.759 |

Table S2：Cancer-related Mortality risk for the four mutually exclusive groups based on MAFLD and sarcopenia

|  | HR | 95% CI | *P* |
| --- | --- | --- | --- |
| **Model 1** |  | | |
| MAFLD-/SARCOPENIA- | **Reference** | | |
| MAFLD+/SARCOPENIA- | 0.851 | 0.696-1.042 | 0.118 |
| MAFLD-/SARCOPENIA+ | 0.895 | 0.767-1.044 | 0.159 |
| MAFLD+/SARCOPENIA+ | 0.927 | 0.795-1.081 | 0.332 |
| **Model 2** |  | | |
| MAFLD-/SARCOPENIA- | **Reference** | | |
| MAFLD+/SARCOPENIA- | 0.850 | 0.666-1.085 | 0.193 |
| MAFLD-/SARCOPENIA+ | 0.871 | 0.724-1.047 | 0.140 |
| MAFLD+/SARCOPENIA+ | 1.000 | 0.828-1.207 | 0.997 |

Results were obtained with Cox proportional hazards and given as HR with 95% CI for all-cause mortality as outcome. Results were adjusted for age and sex (**model 1**) and in addition for race, marital status, education, and smoking (**model 2**).

Abbreviations: CI, confidence interval; HR, hazard ratio; MAFLD, metabolic dysfunction associated fatty liver disease.

Table S3：Fibrosis risk by FIB-4 for the presence of sarcopenia

|  | OR | 95% CI | *P* |
| --- | --- | --- | --- |
| **Model 1** |  |  |  |
| SARCOPENIA | 1.519 | 1.350–1.710 | 0.000 |
| **Model 2** |  |  |  |
| SARCOPENIA | 1.528 | 1.308–1.784 | 0.000 |

Table S4：Fibrosis risk by NFS for the presence of sarcopenia

|  | OR | 95% CI | *P* |
| --- | --- | --- | --- |
| **Model 1** |  |  |  |
| SARCOPENIA | 1.803 | 1.514–2.147 | 0.000 |
| **Model 2** |  |  |  |
| SARCOPENIA | 1.715 | 1.362–2.158 | 0.000 |

Results were obtained with Cox proportional odds and given as OR with 95% CI for all-cause mortality as outcome. Results were adjusted for age and sex (model 1) and in addition for race, marital status, education, and smoking (model 2).

Abbreviations: CI, confidence interval; OR, odd ratio; MAFLD, metabolic dysfunction associated fatty liver disease.

Table S5：Fibrosis risk by FIB-4 for the four mutually exclusive groups based on MAFLD and sarcopenia

|  | OR | 95% CI | *P* |
| --- | --- | --- | --- |
| **Model 1** |  |  |  |
| MAFLD-/SARCOPENIA- | **Reference** | | |
| MAFLD+/SARCOPENIA- | 1.224 | 1.014-1.478 | 0.035 |
| MAFLD-/SARCOPENIA+ | 1.567 | 1.345-1.826 | 0.000 |
| MAFLD+/SARCOPENIA+ | 1.723 | 1.479-2.008 | 0.000 |
| **Model 2** |  |  |  |
| MAFLD-/SARCOPENIA- | **Reference** | | |
| MAFLD+/SARCOPENIA- | 1.104 | 0.865-1.408 | 0.428 |
| MAFLD-/SARCOPENIA+ | 1.600 | 1.307-1.959 | 0.000 |
| MAFLD+/SARCOPENIA+ | 1.647 | 1.342-20.20 | 0.000 |

Table S6：Fibrosis risk by NFS for the four mutually exclusive groups based on MAFLD and sarcopenia

|  | OR | 95% CI | *P* |
| --- | --- | --- | --- |
| **Model 1** |  |  |  |
| MAFLD-/SARCOPENIA- | **Reference** | | |
| MAFLD+/SARCOPENIA- | 1.205 | 0.884-1.643 | 0.239 |
| MAFLD-/SARCOPENIA+ | 1.650 | 1.313-2.073 | 0.000 |
| MAFLD+/SARCOPENIA+ | 2.218 | 1.788-2.752 | 0.000 |
| **Model 2** |  |  |  |
| MAFLD-/SARCOPENIA- | **Reference** | | |
| MAFLD+/SARCOPENIA- | 1.335 | 0.894-1.992 | 0.428 |
| MAFLD-/SARCOPENIA+ | 1.566 | 1.150-2.132 | 0.004 |
| MAFLD+/SARCOPENIA+ | 2.296 | 1.718-3.069 | 0.000 |

Results were obtained with Cox proportional odds and given as OR with 95% CI for all-cause mortality as outcome. Results were adjusted for age and sex (**model 1**) and in addition for race, marital status, education, and smoking (**model 2**).

Abbreviations: CI, confidence interval; OR, odd ratio; MAFLD, metabolic dysfunction associated fatty liver disease.
